# Predictive value of geriatric nutritional risk index in cardiac and cerebrovascular events after endovascular aortic aneurysm repair

**DOI:** 10.3389/fcvm.2024.1399908

**Published:** 2024-10-03

**Authors:** YuPei Zou, Jiarong Wang, Jichun Zhao, Yukui Ma, Bin Huang, Ding Yuan, Yang Liu, Maonan Han, Huatian Gan, Yi Yang

**Affiliations:** ^1^ The Center of Gerontology and Geriatrics, National Clinical Research Center for Geriatrics, West China Hospital, Sichuan University, Chengdu, China; ^2^ Division of Vascular Surgery, Department of General Surgery, West China Hospital, Sichuan University, Chengdu, China

**Keywords:** malnutrition, aged, abdominal aortic aneurysms, endovascular procedures, prognosis

## Abstract

**Objective:**

To evaluate the effect of malnutrition assessed by the Geriatric Nutritional Risk Index (GNRI) on major adverse cardiac and cerebrovascular events (MACCE) in the elderly patients after endovascular aortic aneurysm repair (EVAR).

**Materials and methods:**

This was a retrospective cohort study of elderly patients who underwent EVAR in a tertiary hospital. Malnutrition status was assessed by the GNRI. The primary outcome was MACCE. The predictive ability of the GNRI was compared with both the Revised Cardiac Risk Index (RCRI) and the modified Frailty Index (mFI) using Receiver operating characteristic (ROC) curve.

**Result:**

A total of 453 patients underwent EVAR November 2015 and January 2020 was retrospectively analyzed, equally divided into three (low/medium/high) groups according to GNRI values which ranked from low to high. Five (1.10%) patients were lost in follow-up after surgery, and the median length of follow-up was 28.00 (15.00–47.00) months. The high GNRI values reduced length of hospital stay following EVAR in comparison to patients in low GNRI values group (β 9.67, 95% CI 4.01–23.32, *p* = 0.0113; adjusted β −1.96, 95% CI −3.88, −0.05, *p* = 0.0454). GNRI status was associated with a significantly increased risk of long-term mortality after EVAR (Medium GNRI, unadjusted HR 0.40, 95%CI 0.23–0.70, *p* = 0.0014; adjusted HR 0.47, 95%CI 0.26–0.84, *p* = 0.0107; high GNRI, 0.27 95%CI 0.14–0.55; *p* = 0.0003; adjusted HR 0.32 95%CI 0.15–0.68, *p* = 0.0029). Both medium and high GNRI values were linked to significantly reduced risks of MACCE compared to low GNRI score patients (Medium GNRI, unadjusted HR 0.34, 95%CI 0.13–0.88, *p* = 0.00265; adjusted HR 0.37, 95%CI 0.14–0.96, *p* = 0.0408; High GNRI, 0.26 95%CI 0.09–0.78; *p* = 0.0168; adjusted HR 0.21 95%CI 0.06–0.73, *p* = 0.0029). Compared with the RCRI and mFI, the GNRI had better discrimination in predicting long-term MACCE. An area under the curve (AUC) for GNRI mFI, and RCRI is 0.707, 0.614 and 0.588, respectively. (Z statistic, GNRI vs. mFI, *p* = 0.0475; GNRI vs. RCRI, *p* = 0.0017).

**Conclusion:**

Malnutrition assessed by the GNRI may serve as a useful predictor of long-term MACCE in elderly patients after EVAR, with preferable discrimination abilities compared with both RCRI and mFI.

## Introduction

Endovascular aortic aneurysm repair (EVAR) is the preferred intervention for abdominal aortic aneurysm (AAA), particularly in elderly patients with multiple comorbidities. Despite its minimally invasive procedures, EVAR patients remain susceptible to various adverse outcomes, including cardiac and cerebrovascular events (1, 2) with complication rates ranging from 0.94% to 4.5% (3, 4) during follow-up. Given the potentially profound impact of cardiovascular events on patient quality of life and survival, there is a critical need for robust predictive models to aid preoperative assessment and postoperative monitoring. While tools like the Modified Frailty Index (mFI) (5) and the Revised Cardiac Risk Index (RCRI) (6) have emerged in recent years, we previously found adverse event incidence, exhibiting significant variability across different risk calculators (7). Moreover, existing risk values primarily address short-term cardiovascular complications, with a dearth of predictors for long-term major adverse cardiac and cerebrovascular events (MACCE).

Above tools primarily evaluate the physiological condition based on comorbidities to forecast surgical risks and prognosis, with limited consideration for the nutritional status of patients. The Geriatric Nutritional Risk Index (GNRI) (8) serves as a tailored nutritional assessment tool for elderly patients, specifically aimed at gauging the deleterious effects of nutritional elements on clinical endpoints. Nutritional risk among hospitalized elderly patients may lead to prolonged hospitalization, heightened healthcare expenditures, increased perioperative complications, and elevated mortality rates. GNRI has demonstrated significant associations with postoperative complications and prognostic outcomes across a spectrum of diseases (9–11). Our prior meta-analysis revealed that the validation of most frailty assessment instruments predominantly focuses on short-term survival outcomes post-vascular surgery, highlighting a notable deficiency in tools of high quality addressing both short-term and long-term cardiovascular endpoints (7).

Therefore, our study sought to ascertain the predictive efficacy of the GNRI concerning short-term and long-term outcome as well as adverse cardiovascular events in patients undergoing EVAR.

## Materials and methods

### Ethics

This retrospective cohort study was conducted at West China Hospital, a tertiary academic center in Chengdu, Sichuan, China. It adhered to the Strengthening the Reporting of Observational Studies in Epidemiology (STROBE) guidelines for cohort studies. The study protocol received approval from the institutional review board of West China Hospital, with a waiver of informed consent.

### Selection and description of participants

Consecutive individuals aged over 60 years who underwent EVAR at West China Hospital between November 2015 and January 2020 were screened for eligibility. Exclusion criteria comprised patients lacking functional status records, readmitted individuals receiving reinterventions for prior EVAR, those with incorrect contact information, and individuals unable to communicate. Data for patients admitted were retrospectively gathered from our EVAR database, followed by prospective data collection thereafter. Patients underwent outpatient follow-up with Duplex ultrasound at 1, 6, and 12 months post-intervention, and subsequently annually. Telephone follow-up was utilized if patients failed to attend appointments. Any observed adverse events, such as endoleaks or limb thrombosis, during Duplex ultrasound examinations prompted further evaluation via computed tomography angiogram.

### GNRI identification

This study compiled preoperative patient data that was available in the electronic medical record system, encompassing demographic variables such as gender and age, as well as medical histories pertaining to smoking, hypertension, diabetes, lung disease, heart disease, and other comorbidities, alongside perioperative mortality and complication rates. Additionally, measurements including height, body weight, body mass index (BMI), and serum protein levels were documented. The nutritional status of all patients was assessed using the Geriatric Nutritional Risk Index (GNRI) formula (8). GNRI = (14.89 × serum albumin level g/dl) + (41.7 × current body weight ÷ ideal body weight); Ideal body weight = height(m) × height(m) × 22. According to the calculated GNRI values, 453 patients included in this study were divided equally into three groups. Charlson Comorbidity Index (CCI) (12), RCRI (6) and mFI values (4, 5, 13) were also calculated for each patient according to previous report. Another baseline demographic and clinical parameters were gathered, including emergent case classification, severely angulated neck, concurrent common iliac artery aneurysm (CIAA) with a maximum diameter ≥25 mm, and maximum diameter of abdominal aortic aneurysm (AAA) (expressed in mm).

In this study, the primary outcomes evaluated encompassed short-term and long-term major adverse cardiac and cerebrovascular events (MACCE).MACCE was defined as a composition of death, myocardial infarction, stroke, chronic cardiac failure and repeat revascularization (14) Secondary outcomes included length of stay in hospital, 30-day mortality, overall survival, and adverse aortic events (AAE). AAE was defined as a composition of type I or III endoleaks, limb occlusion, and aortic-related reintervention.

### Statistical analysis

Continuous variables were depicted as means ± standard deviations or as medians with interquartile ranges in cases of non-normal distribution. Categorical variables were articulated as numbers and percentages. Comparative analyses among the three groups involved ANOVA or Kruskal–Wallis H tests for continuous variables and *χ*^2^ or Fisher's exact tests for categorical variables. Long-term outcomes were delineated using absolute frequencies (patient count) and relative frequencies (percentages), with proportions presented alongside 95% confidence intervals (CI). Time-to-event data rates were computed using the Kaplan–Meier method. Receiver operating characteristic (ROC) curves were generated to evaluate the sensitivity, specificity, and area under the curve (AUC) of mFI, RCRI and GNRI in predicting the outcome of interest. The optimal cutoff value was determined based on the maximum value of the Youden index. Statistical differences in the area under the ROCs were compared using the Delong's method (15).

Univariate analysis employing the log-rank test evaluated the correlation between GNRI and the outcomes of interest. Cox proportional hazard regression analysis was utilized to determine adjusted hazard ratios (HR) and corresponding 95% CI for long-term outcomes. Multivariate logistic regression was employed to compute adjusted odds ratios (OR) and 95% CI for short-term outcomes. Covariates integrated into the multivariate analyses were selected based on their impact on outcome measures during univariate analysis, wherein their inclusion altered the HR or OR by at least 10%. Baseline age, gender, CCI and emergent case were also included in the adjustment. Considering GNRI as a composite measure of nutrition risk, incorporating functional status and health conditions within its causal pathway, we refrained from adjusting for these factors in our analysis. All statistical analyses were performed using IBM SPSS Statistics Version 29.0.2.0 (IBM Corp, Armonk, NY), MedCalc® Statistical Software version 22.017 (MedCalc Software Ltd, Ostend, Belgium; https://www.medcalc.org; 2024), and R studio Version.

1.2.1335 (http://www.R-project.org/).

## Results

### Baseline characteristics

**Figure 1:**
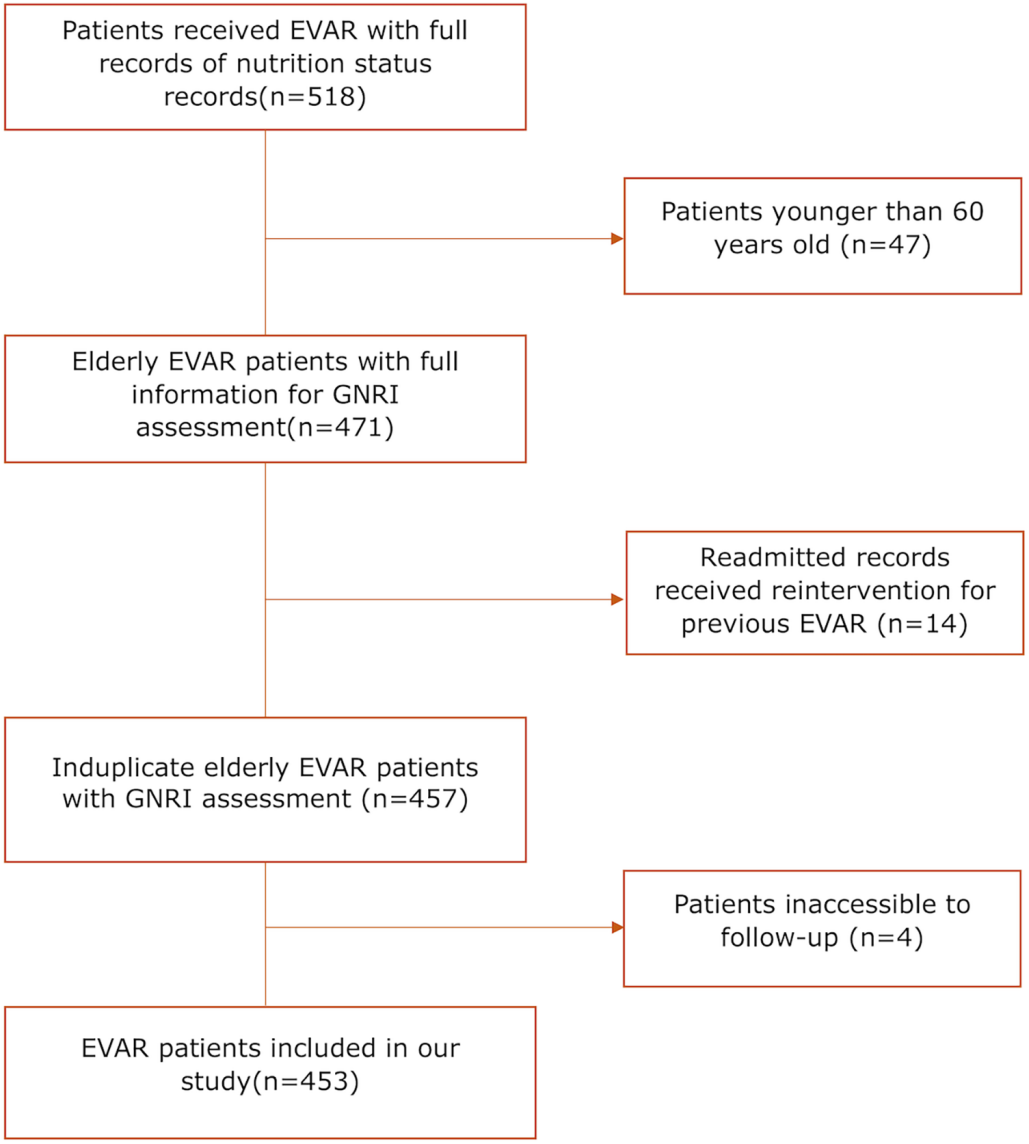
Flow diagram of patient inclusion and exclusion; EVAR, endovascular aortic repair; GNRI, geriatric nutritional risk Index.

453 patients who underwent EVAR from November 2015 to January 2020 in West China Hospital were finally retrospective analyzed in our study, with detailed flow diagram shown in Figure 1. The median age of this cohort was 73 (67–78) years, and 85.21% of patients were male. Based on the calculated GNRI values ranking from low to high, these patients were divided equally into three groups, with 151 patients in each group. The median value was 102.03 (95.34–109.83). The CCI score was also calculated for each patient, with 48.34% of 0 point, 30.68% of 1 point, 10.38% of 2 points, 6.84% of 3 points 1.99% of 4 points, 1.33% of 5 points and 0.44% of 6 points. Five (1.10%) patients were lost in follow-up after surgery, and the median length of follow-up was 28.00 (15.00–47.00) months. The detailed baseline characteristics of low, medium and high GNRI values of patients were shown in Table 1.

**Table 1:** Baseline characteristics of patients with different GNRI statuses.

GNRI tertile	Low	Medium	High	*P*-value
*N*	151	151	151	
Age-year	74.88 ± 7.20	72.49 ± 7.95	70.25 ± 8.19	<0.001^a^
Weight-kg	55.97 ± 8.93	63.09 ± 8.49	73.07 ± 9.99	<0.001^a^
Height-m	1.66 ± 0.07	1.66 ± 0.07	1.66 ± 0.07	0.797
BMI	20.30 ± 2.63	22.94 ± 2.29	26.36 ± 3.09	<0.001^a^
ALB-g/dl	34.50 ± 4.28	39.37 ± 2.99	44.95 ± 9.27	<0.001
GNRI	89.85 ± 6.75	102.11 ± 2.67	116.89 ± 13.58	<0.001^a^
Gender				0.523
Male	125 (82.78%)	129 (85.43%)	132 (87.42%)	
Female	26 (17.22%)	22 (14.57%)	19 (12.58%)	
Smoking	98 (64.90%)	91 (60.26%)	91 (60.26%)	0.632
Hypertension	93 (61.59%)	98 (64.90%)	121 (80.13%)	0.001^a^
Diabetes	10 (6.62%)	24 (15.89%)	23 (15.23%)	0.025^a^
Pulomary	40 (26.49%)	32 (21.19%)	19 (12.58%)	0.01^a^
Stroke	3 (1.99%)	11 (7.28%)	8 (5.30%)	0.096
CAD	30 (19.87%)	27 (17.88%)	32 (21.19%)	0.767
CKD	13 (8.61%)	4 (2.65%)	5 (3.31%)	0.031^a^
Emergency	22 (14.57%)	18 (11.92%)	20 (13.25%)	0.794
General anesthesia	54 (35.76%)	46 (30.46%)	30 (19.87%)	0.008^a^
Length of stay-d	13.87 ± 9.50	12.27 ± 8.10	11.39 ± 7.67	0.037^a^
30-day death	3 (1.99%)	0	1 (0.66%)	0.605
SNA	48 (31.79%)	45 (29.80%)	29 (19.21%)	0.03^a^
RCRI	1.36 ± 0.65	1.33 ± 0.55	1.38 ± 063	0.794
mFI	1.60 ± 1.22	1.55 ± 1.21	1.47 ± 1.05	0.614
CCI	0.96 ± 1.16	0.91 ± 1.17	0.81 ± 1.16	0.514
Neck diameter	21.29 ± 2.85	21.29 ± 2.95	21.40 ± 2.67	0.934
Neck length	27.94 ± 13.23	26.04 ± 12.24	30.30 ± 14.53	0.06^a^
Maximum diameter	56.01 ± 13.86	55.11 ± 13.57	51.88 ± 12.72	0.02^a^

BMI, body mass index; CAD, coronary artery disease; CCI, Charlson complication index; CIAA, common iliac artery aneurysm; CKD, chronic kidney disease; COPD, chronic obstructive pulmonary diseases; GNRI, geriatric nutritional risk index; SNA, severe neck angulation. Data are presented as number (%) or mean ± stand deviation.

^a^ Statistically significant.

### Length of stay in hospital and 30-day mortality

Two of these patients (2/453, 0.44%) died in hospital postoperatively, from heart failure and pulmonary infection. The cohort exhibited an overall 30-day mortality rate of 0.88% (4/453), for low/medium/high GNRI values groups were 1.99%, 0.00% and 0.66%, respectively. The median length of stay in hospital was 8 (11–14) days. The mean length of hospital stay was significantly different in three groups. Both univariate and multivariate analyses indicated a notable association between high GNRI values and reduced length of hospital stay following EVAR in comparison to patients with low GNRI values (β 9.67, 95% CI 4.01–23.32, *p* = 0.0113; adjusted β −1.96, 95% CI −3.88, −0.05, *p* = 0.0454),but not with 30-day mortality listed in Table 2.

**Table 2:** Results of regression analysis of main short-term outcomes.

Exposure	Non-adjusted	Adjust I^a^	Adjust II^b^
Length of stay	β (95%CI) *P*-value	β (95%CI) *P*-value	β (95%CI) *P*-value
GNRI tertile			
Low	Reference	Reference	Reference
Medium vs. Low	−1.60 (−3.50, 0.31) 0.1017	−1.67 (−3.59, 0.26) 0.0900	−1.55 (−3.40, 0.29) 0.0997
High vs. Low	−2.48 (−4.38, −0.57) 0.0113	−2.60 (−4.56, −0.64) 0.0097	−1.96 (−3.88, −0.05) 0.0454
GNRI > 99 vs. ≤99	−1.82 (−3.42, −0.22) 0.0261	−1.91 (−3.54, −0.28) 0.0222	−1.68 (−3.27, −0.09) 0.0392
30-day mortality	OR (95%CI) *P*-value	OR (95%CI) *P*-value	OR (95%CI) *P*-value
GNRI tertile			
Low	Reference	Reference	Reference
Medium vs. Low	NA	NA	NA
High vs. Low	0.32 (0.03, 3.15) 0.3320	0.29 (0.03, 3.13) 0.3049	0.47 (0.04, 5.98) 0.5615
GNRI > 99 vs. ≤99	0.20 (0.02, 1.99) 0.1713	0.19 (0.02, 1.89) 0.1549	0.26 (0.02, 2.85) 0.2731

GNRI, geriatric nutritional risk index; CI, confidence interval; CCI, Charlson complication index; OR, odds ratios.

^a^ Adjust I model adjust for: GENDER; AGE.

^b^ Adjust II model adjust for: GENDER; AGE; CCI; EMERGENT.

### Overall survival

**Figure 2:**
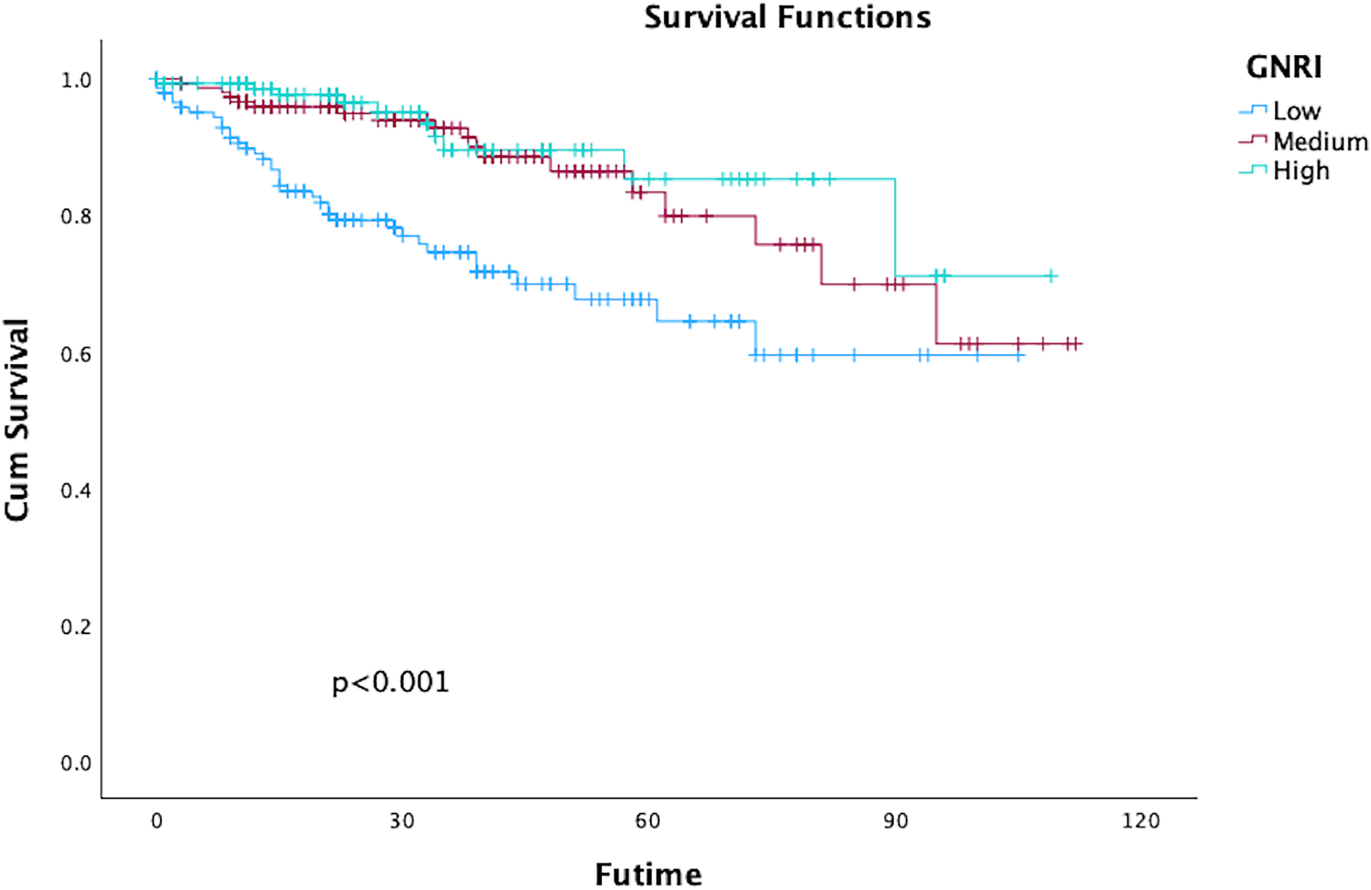
Kaplan–Meier curves of survival in patients with the different geriatric nutritional risk Index (GNRI) values after endovascular aortic repair (EVAR). (Log-rank test, *P* < 0.001).

The rate of overall survival in 1-,3-,5-years during following up were 99.1%, 97.7%, and 95.6%, respectively, GNRI status significantly impacted on long-term all-cause death which was outlined in Figure 2; Table 3. Both univariate and multivariate regression analyses indicated a significantly heightened risk of long-term mortality associated with low GNRI values. (Medium GNRI, unadjusted HR 0.40, 95%CI 0.23–0.70, *p* = 0.0014; adjusted HR 0.47, 95%CI 0.26–0.84, *p* = 0.0107; High GNRI, 0.27 95%CI 0.14–0.55; *p* = 0.0003; adjusted HR 0.32 95%CI 0.15–0.68, *p* = 0.0029). Up to five years, survival for low, medium and high GNRI score patients were 79.2%, 92.7%, 96.5%, respectively.

**Table 3:** Results of regression analysis of main long-term outcomes.

Exposure	Non-adjusted	Adjust I^a^	Adjust II^b^
Overall survival	HR (95%CI) *P*-value	HR(95%CI) *P*-value	HR(95%CI) *P*-value
GNRI tertile			
Low	1	1	1
Medium vs. Low	0.40 (0.23, 0.70) 0.0014	0.47 (0.27, 0.83) 0.0094	0.47 (0.26, 0.84) 0.0107
High vs. Low	0.27 (0.14, 0.55) 0.0003	0.37 (0.18, 0.76) 0.0063	0.32 (0.15, 0.68) 0.0029
*GNRI > 99* vs. *≤99*	0.39 (0.22, 0.59) < 0.0001	0.43 (0.26, 0.71) 0.001	0.40 (0.24, 0.68) 0.0007
Reintervention			
GNRI tertile			
Low	1	1	1
Medium vs. Low	0.77 (0.30, 1.95) 0.5824	0.76 (0.30, 1.96) 0.5767	0.78 (0.29, 2.05) 0.6078
High vs. Low	1.11 (0.44, 2.81) 0.8227	1.08 (0.41, 2.82) 0.8804	1.09 (0.40, 2.94) 0.8646
*GNRI > 99* vs. *≤99*	0.98 (0.45, 2.16) 0.9692	0.98 (0.44, 2.17) 0.9589	0.99 (0.44, 2.24) 0.9768
AAE			
GNRI tertile			
Low	1	1	1
Medium vs. Low	0.51 (0.12, 2.12) 0.3509	0.57 (0.13, 2.42) 0.4430	0.59 (0.14, 2.53) 0.4739
High vs. Low	0.22 (0.03, 1.89) 0.1675	0.26 (0.03, 2.29) 0.2234	0.28 (0.03, 2.56) 0.2602
*GNRI > 99* vs. *≤99*	0.49 (0.13, 1.81) 0.2822	0.56 (0.15, 2.12) 0.3904	0.54 (0.14, 2.10) 0.3817
MACCE			
GNRI tertile			
Low	1	1	1
Medium vs. Low	0.34 (0.13, 0.88) 0.0265	0.35 (0.13, 0.91) 0.0308	0.37 (0.14, 0.96) 0.0408
High vs. Low	0.26 (0.09, 0.78) 0.0168	0.27 (0.09, 0.84) 0.0239	0.21 (0.06, 0.73) 0.0141
*GNRI > 99* vs. *≤99*	0.28 (0.18, 0.44) < 0.0001	0.31 (0.19, 0.50) < 0.0001	0.26 (0.16, 0.43) < 0.0001

AAE, adverse aortic events; CI, confidence interval; CCI, Charlson complication index; HR, hazard ratio; GNRI, geriatric nutritional risk index; MACCE, major adverse cardiac and cerebrovascular events.

^a^ Adjust I model adjust for: GENDER; AGE

^b^ Adjust II model adjust for: GENDER; AGE; CCI; EMERGENT.

### Long-term major adverse cardiac and cerebrovascular events

**Figure 3:**
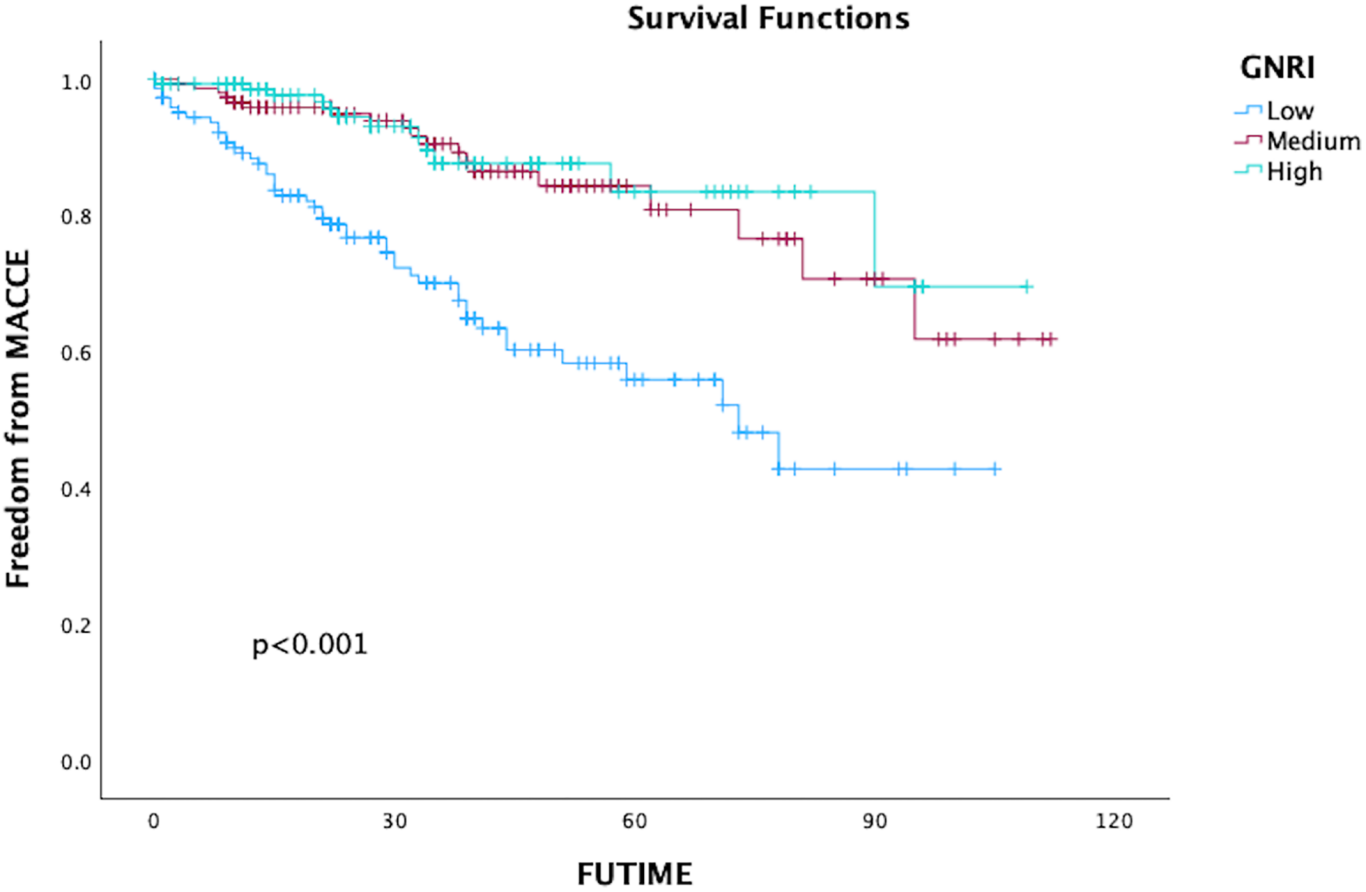
Kaplan–Meier curves of freedom of MACCE in patients with different geriatric nutritional risk Index (GNRI) status after endovascular aortic repair (EVAR).

**Figure 4:**
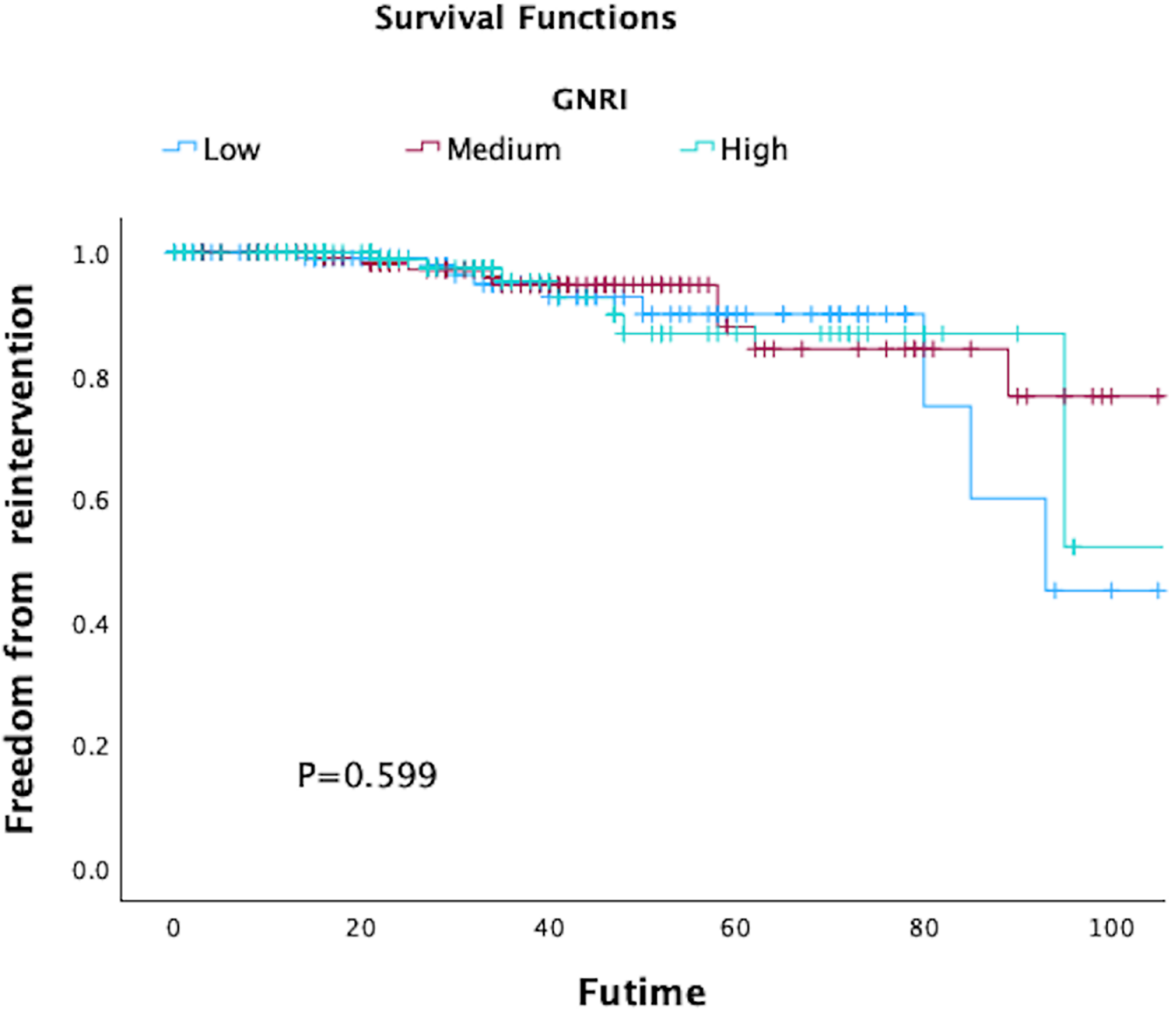
Kaplan–Meier curves of freedom of reintervention in patients with different geriatric nutritional risk Index (GNRI) status after endovascular aortic repair (EVAR).

In total, 80 patients (17.66%) experienced MACCE during the follow-up period, comprising 63 deaths, 12 adverse cardiac events, and 8 strokes. The five-year freedom from MACCE rates were 78.7.2% for low GNRI score patients, 93.9% for medium score patients, and 96.7% for high GNRI patients. The findings from both univariate and multivariate analysis indicated that both medium and high GNRI values were linked to significantly decreased risks of Major Adverse Cardiac and Cerebrovascular Events (MACCE) compared to low GNRI score patients (Medium GNRI, unadjusted HR 0.34, 95%CI 0.13–0.88, *p* = 0.00265; adjusted HR 0.37, 95%CI 0.14–0.96, *p* = 0.0408; High GNRI, 0.26 95%CI 0.09–0.78; *p* = 0.0168; adjusted HR 0.21 95%CI 0.06–0.73, *p* = 0.0029, Table 3). The freedom from MACCE was listed in Figure 3. During follow-up, 27 (5.96%) of patients had reinterventions, due to 8 (1.76%) type IA endoleaks, 7 (1.54%) type IB endoleaks, 3 (0.66%) type II endoleaks and 5 (1.10%) limb occlusions. The rate of freedom from reinterventions at three years in low, medium and high GNRI values patients were 97.8%, 97.2%, 98.9%, respectively (Figure 4). Various GNRI status did not demonstrate significant associations with aortic-related mortality or reintervention (Table 3).

### Comparison of performance measures of both mFI and RCRI with GNRI

**Figure 5:**
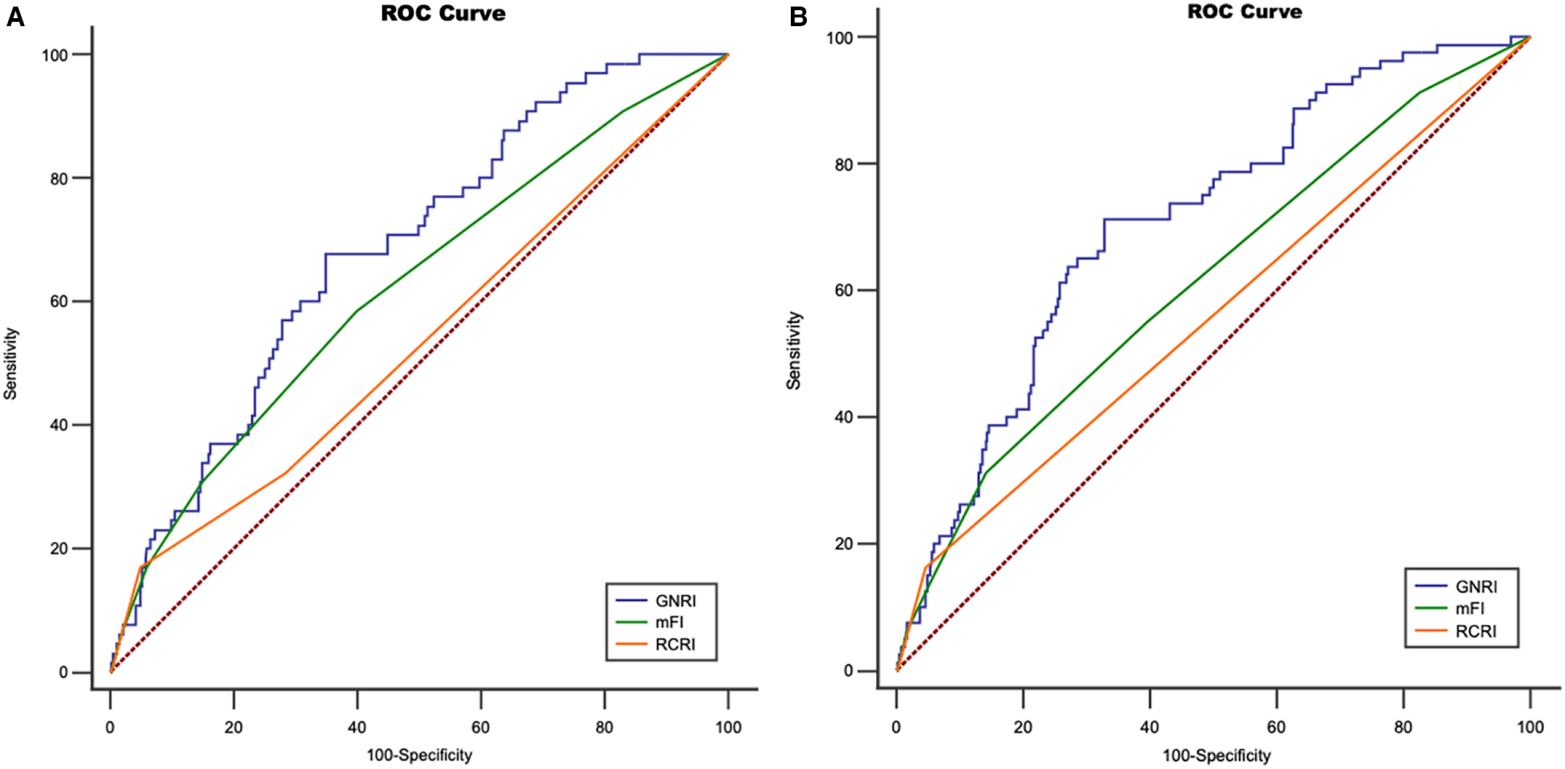
Receiver operating characteristic curve for (ROC) **(A)** geriatric nutritional risk index (GNRI), modified frailty index index(mFI) and revised cardiac risk Index (RCRI) regarding major adverse cardiac and cerebrovascular events (MACCE), An area under the curve (AUC) for GNRI mFI, and RCRI is 0.707, 0.614 and, 0.588, respectively. (Z statistic, GNRI vs. mFI, *p* = 0.0475; GNRI vs. RCRI, *p* = 0.0017; mFI vs. RCRI, *p* = 0.0423); **(B)** ROC for GNRI, mFI and RCRI regarding long-term survival, AUC for GNRI, mFI, and RCRI is 0.683, 0.622, and 0.535, respectively. (Z statistic, GNRI vs. mFI, *p* = 0.2377; GNRI vs. RCRI, *p* = 0.0056; mFI vs. RCRI, *p* = 0.039).

As shown in the ROC curve (Figure 5A), late MACCE was set as state variable. c-statistic (area under curve) was 0.707, 95% CI 0.662–0.749. The Area Under the Curve (AUC) analysis indicated superior discriminatory ability of the GNRI for MACCE compared to other assess tool including mFI and RCRI Figure 5A. When considering death as a competing risk, both GNRI (AUC 0.683, 95% CI 0.638–0.749) and mFI (AUC 0.622, 95% CI 0.575–0.667) showed similar better discriminatory ability for long-term survival compared to RCRI (AUC 0.535, 95% CI 0.488–0.582) (Figure 5B).

**Figure 6:**
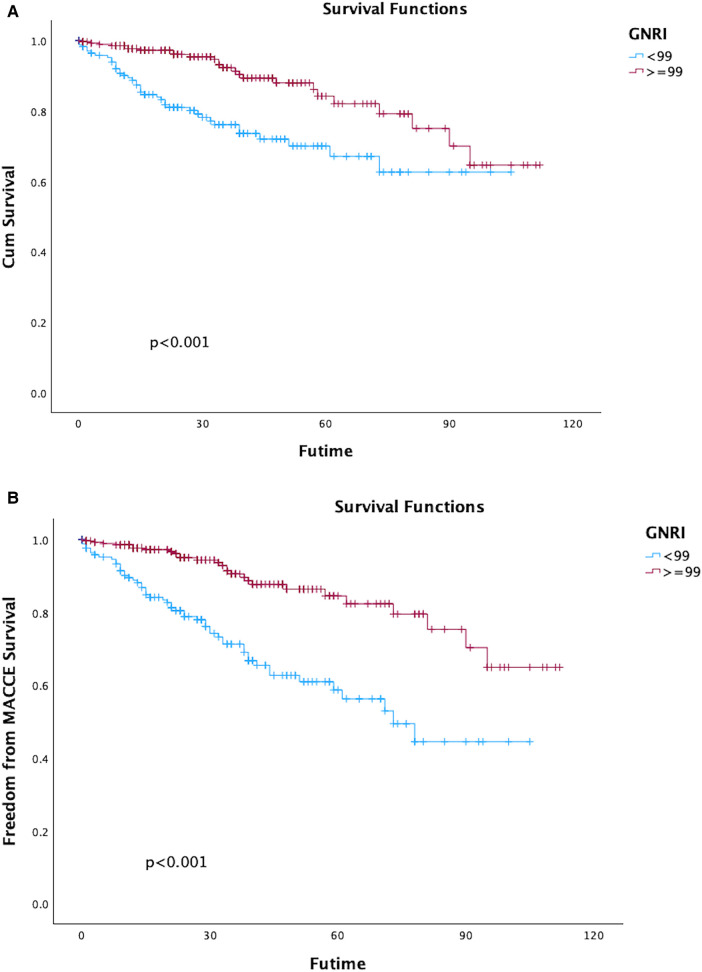
Kaplan–Meier curves of survival **(A)** and freedom of major adverse cardiac and cerebrovascular events (MACCE) **(B)** in patients with geriatric nutritional risk index (GNRI) ≥ 99 vs. <99 after endovascular aortic repair (EVAR).

Nevertheless, the GNRI showed a discernible impact on outcomes in long-term MACCE after EVAR. Hence, it is imperative to determine a cutoff value for GNRI to enhance its utility in guiding clinical practice, the GNRI value = 99 was selected as cutoff value with maximum discriminative power (sensitivity 71.3%, specificity 66.9%). 278 patients (61.3%) had GNRI ≥ 99, 175 patients had GNRI < 99. Significant differences were observed in all-cause mortality and long-term MACCE between the two groups, showed in Figure 6; Tables 2, 3.

## Discussion

This cohort study represented the first investigation into the impact of malnutrition status assessed by GNRI on long-term MACCE in patients following EVAR, while also assessing the predictive capacity of the GNGI tentatively compared to both RCRI and mFI. Our study indicated a significant association between low GNRI and extended perioperative hospitalization, elevated long-term all-cause mortality, and long-term MACCE in patients following EVAR. Moreover, our result suggested that may offer superior predictive capability for long-term MACCE and survival in patients undergoing EVAR compared to RCRI score.

The growing utilization of minimally invasive endovascular therapies for AAA highlighted the imperative need to identify patients considered ineligible for surgery (16). While cardiovascular, pulmonary, and renal diseases are common risk factors associated with heightened surgical risk, emerging studies indicate that diminished physiologic reserves, influenced by frailty, malnutrition, sarcopenia, cognitive impairment, and other factors, may diminish the ability to recuperate from surgical stresses (16–18). As cardiac complications can lead to over 40% of perioperative deaths after non-cardiac surgery (19), the contemporary AAA guidelines respectively developed by the European and American Society for Vascular Surgery pointed out the significance of perioperative cardiac risk assessment (20, 21). Apart from postoperative cardiac factors, long-term MACCE could significantly affect the prognosis of patients after EVAR during following-up, especially in the elderly with multiple comorbidities. Furthermore, current predictive models after vascular surgery have been found to underestimate short-term cardiovascular risk (3). There are no recognized predictive tools that are effective in predicting long-term MACCE, and there is a need to develop appropriate predictive tools for predicting short/long-term prognosis in patients undergoing EVAR (7).

Elderly individuals are prone to malnutrition (22). The GNRI, which is an assessment tool for nutritional status, has been utilized as a prognostic factor for complications and mortality in elderly patients (8). Several studies have identified poorer postoperative outcomes in abdominal surgery among patients with a low GNRI (8, 23). Limited data existed regarding the association between the GNRI and the postoperative prognosis of EVAR. NISHIBE (24) reported that the GNRI may serve as the more precise nutritional indicator for identifying a potentially high-risk group for mortality following Endovascular Aneurysm Repair EVAR, compared to both ALB and BMI. Another study illustrated that assessing both sarcopenia and nutritional status could forecast late mortality in patients undergoing EVAR (25). These studies solely concentrated on the adverse impact of the GNRI on overall mortality, without examining its predictive ability for MACCE. Similarly, our study found that the patients in the high GNRI group had a shorter hospital stay compared to patients in the low GNRI group, with an average reduction of 1.96 days. (95% CI, −3.88, −0.05, *p* = 0.0454) and the GNRI could predict long term survival up to 5 years. These results enhance the credibility of GNRI as a predictor for long-term outcomes following EVAR. In contrast to MACCE and overall survival, our findings indicate that the GNRI may exhibit limited predictive value for aortic-related mortality, or reintervention following EVAR. This could be attributed to the current nutrition assessment methods predominantly reflecting systemic conditions rather than anatomical features. In the original study, patients were stratified into four groups based on their GNRI values (8). In our investigation, patients were categorized into three groups based on their GNRI levels (low, medium, and high). Our findings also indicated that GNRI served as a robust predictor of overall long-term survival and risk of MACCE. Previous report suggested that the GNRI could serve as a prognostic predictor following both abdominal surgery and EVAR at 98 cutoff value (23, 25). We employed an approximate cutoff value of 99 to identify patients meeting the criteria for malnutrition.

The RCRI, a traditional cardiac risk score in vascular surgery, demonstrated an independent predictive effect on long-term major adverse cardiac events following carotid endarterectomy (26). Prior studies suggested that frailty evaluated by the mFI could effectively predict both short-term and long-term MACCE in elderly patients following EVAR, with enhanced discrimination and reclassification capabilities compared to the RCRI (4). In this investigation, we initially assessed the predictive capabilities of GNRI, mFI, and RCRI for long-term overall survival and MACCE using the area under the ROC curve. GNRI exhibited slightly higher AUC values compared to mFI, and demonstrated superior discrimination abilities in comparison to RCRI. Following treatment guidelines, EVAR has emerged as the primary therapeutic approach for elderly patients with high surgical risk abdominal aortic aneurysms at our institution. Previous study reported that EVAR did not alter the long-term overall survival of patients with frailty (27). It may be feasible to contemplate strategies such as expanding the aneurysm diameter as a surgical criterion or opting for non-surgical observation for patients exhibiting both frailty and malnutrition, which are closely associated with adverse outcomes post-intervention. Our upcoming investigation will concentrate on quantitatively evaluating the balance between aneurysm rupture risk and postoperative adverse events, integrating assessments of frailty index and nutritional status.

Our study has several limitations. First, this was a single-center retrospective study, characterized by selection bias towards EVAR. EVAR-treated patients were older and presented with more comorbidities, making them more prone to meeting the criteria for malnutrition. It cannot establish a causal relationship between malnutrition and MACCE or mortality. Second, a single preoperative measurement of GNRI may not entirely reflect a patient's nutritional status; changes in a patient's condition could have occurred between the nutrition assessment, operation, or follow-up, potentially remaining undetected. Third, the median follow-up time of 33 months in our study was relatively short, and longer field follow-up studies are needed in the future to further validate the predictive value of the GNRI. Finally, we did not perform external validation for comparison of these predictive models.

## Conclusion

This cohort study suggested that malnutrition assessed by the GNRI may have significant effects on both long-term mortality and MACCE in elderly patients after EVAR. Further, the GNRI may serve as a better predictive tool for MACCE compared to the RCRI. This assessment tool could be useful for risk stratification of long-term MACCE in the elderly patients after EVAR. Enhanced cardiovascular risk management and stricter surveillance plan should be considered for malnutrition patients after EVAR.

## Data Availability

The raw data supporting the conclusions of this article will be made available by the authors, without undue reservation.
